# Survival in Women with De Novo Metastatic Breast Cancer: A Comparison of Real-World Evidence from a Publicly-Funded Canadian Province and the United States by Insurance Status

**DOI:** 10.3390/curroncol29010034

**Published:** 2022-01-16

**Authors:** Marie-France Savard, Elizabeth N. Kornaga, Adriana Matutino Kahn, Sasha Lupichuk

**Affiliations:** 1 The Ottawa Hospital Cancer Centre, Ottawa, ON K1H 8L6, Canada; 2 Tom Baker Cancer Centre, Calgary, AB T2N 4N2, Canada; elizabeth.kornaga@albertahealthservices.ca (E.N.K.); Sasha.lupichuk@albertahealthservices.ca (S.L.); 3 Department of Medicine, Hematology Oncology, Yale New Haven Hospital, New Haven, CT 06510, USA; adriana.kahn@yale.edu

**Keywords:** overall survival, breast neoplasm, insurance status, United States, Canada

## Abstract

Metastatic breast cancer (MBC) patient outcomes may vary according to distinct health care payers and different countries. We compared 291 Alberta (AB), Canada and 9429 US patients < 65 with de novo MBC diagnosed from 2010 through 2014. Data were extracted from the provincial Breast Data Mart and from the National Cancer Institute’s SEER program. US patients were divided by insurance status (US privately insured, US Medicaid or US uninsured). Kaplan-Meier and log-rank analyses were used to assess differences in OS and hazard ratios (HR) were estimated using Cox models. Multivariate models were adjusted for age, surgical status, and biomarker profile. No difference in OS was noted between AB and US patients (HR = 0.92 (0.77–1.10), *p* = 0.365). Median OS was not reached for the US privately insured and AB groups, and was 11 months and 8 months for the US Medicaid and US uninsured groups, respectively. The 3-year OS rates were comparable between US privately insured and AB groups (53.28% (51.95–54.59) and 55.54% (49.49–61.16), respectively). Both groups had improved survival (*p* < 0.001) relative to the US Medicaid and US uninsured groups [39.32% (37.25–41.37) and 40.53% (36.20–44.81)]. Our study suggests that a universal health care system is not inferior to a private insurance-based model for de novo MBC.

## 1. Introduction

In North America, it is estimated that one out of eight women will develop breast cancer during their lifetime [1]. Of these women, about 5% will be diagnosed with de novo stage IV (metastatic) disease [2]. Median and 5-year relative survival amongst patients with de novo stage IV breast cancer have been increasing [3,4]. However, there is significant variability in outcomes for this group of patients. Intrinsic tumor-related factors, such as triple negative status, and extrinsic factors, such as older age, African American ethnicity, and low socioeconomic status and/or lack of health insurance, have been associated with poor prognosis [2,5].

In the United States (US), health insurance is either privately funded or government-funded, through Medicaid and Medicare, for low-income individuals and those ≥ 65 years, respectively [6]. There is substantial variability in health care coverage amongst the different insurance policies [7]. Further, despite the Affordable Care Act (ACA) becoming law in 2010, a significant proportion of individuals remain uninsured. In Canada, access to health care is considered universal and based on a public payer model managed by the respective provinces, including the domain of cancer care. However, the availability of new systemic cancer therapies may differ in scope or be delayed in comparison to the US [8,9]. Health Canada approvals are slower compared with the FDA [10]. In addition, new systemic therapies must be vetted by a national body for clinical benefit and cost-effectiveness [11,12]. Only recommended drugs can then be considered for provincial funding.

In this retrospective study, we sought to benchmark survival outcomes of patients with de novo metastatic breast cancer (MBC) in Alberta (AB), Canada through comparison to a similar cohort in the US as a whole and by insurance status (US privately insured, US Medicaid or US uninsured).

## 2. Materials and Methods

### 2.1. Data Sources and Patient Selection

Population-based data for the AB cohort was obtained from the Alberta Health Services Cancer Control Breast Data Mart (BDM). The BDM is a repository of information on all patients diagnosed with breast cancer from 1 January 2004 onwards in the province of Alberta, Canada. The US cohort was obtained from the National Cancer Institute’s Surveillance, Epidemiology, and End Results (SEER) program November 2018 submission, released April 2019. The SEER program is a data repository that has been collecting population-based information within the US since 1 January 1973, and which currently includes 20 cancer registries covering approximately 34.6% of the population [13].

Female patients 18–64 years of age who were diagnosed with de novo MBC from 1 January 2010 to 31 December 2014 where included. We did not include patients diagnosed prior to 2010 as human epidermal growth factor receptor-2 (HER2) status was not mandatorily reported to cancer registries. Further, we did not include patients 65 or older due to unreliable insurance status classification in the SEER registry. The following data were extracted from the BDM and SEER for all eligible patients: demographics (age at diagnosis and date of diagnosis), clinical-pathological factors (whether or not patient underwent primary surgery, i.e., breast conservation or mastectomy, hormone receptor [HR] status positive versus negative, and HER2 status positive versus negative), and vital status with date of death where applicable.

### 2.2. Statistical Analysis

Women less than 65 years of age from the US cohort were grouped by their insurance status into US privately insured, US Medicaid or US uninsured, with the US privately insured set as the reference group. Cohorts were further categorized by age group (18–49 and 50–64), surgical status (surgery vs. no surgery), and biomarker profile (HR+/HER2−, HER2+, and triple negative [TN] defined as HR−/HER2−).

All statistical analyses were performed using Stata (version 12.1; StataCorp, College Station, TX, USA). 3-year overall survival (OS) was the outcome of interest and median OS was determined where possible. Comparison of clinical-pathological variables between cohorts utilized Wilcoxon’s rank sum and Pearson’s Χ^2^, excluding cases with missing data. Kaplan-Meier and log-rank analyses were used to assess differences in 3-year OS, and hazard ratios (HR) were calculated using the Cox proportional hazard regression model. Multivariate models were adjusted for surgery status, age (continuous) and biomarker profile. All *p*-values were two-sided, and *p* < 0.05 was considered statistically significant.

## 3. Results

### 3.1. US and AB Cohorts

A total of 9492 cases were included in the US cohort and 291 cases in the AB cohort (Table S1). The US cohort was slightly older than the AB cohort, median age 54 years compared with 52 years [*p* = 0.029]. There were no significant differences in the distribution of the cohorts by age group (18–49 vs. 50–64), year of diagnosis, or receipt of primary surgery. There were differences seen with regards to distribution by biomarker profile between the US and AB cohorts (*p* = 0.012). Specifically, in the US cohort relative to the AB cohort, the proportion of TN breast cancer was higher (12.9% vs. 8.6%), and there were lower proportions of both HR+/HER2− breast cancer (49.2% vs. 60.5%), and HER2+ breast cancer (25.1% vs. 30.9%). However, a total of 1213 women (12.8%) in the US cohort did not have biomarker information available.

Median OS in the US cohort was 35 months and not reached for the AB cohort (log-rank *p* = 0.0127). The 3-year OS was slightly lower in the US cohort compared to the AB cohort (48.67% (47.59–49.74) and 55.54% (49.49–61.16), respectively, *p* = 0.0127). Women in the AB group appeared to have a lower risk of mortality than the US cohort (HR = 0.80 (0.67–0.96), *p* = 0.014); however, no difference was noted between the cohorts after adjustment for age, receipt of primary surgery, and biomarker profile (HR = 0.92 (0.77–1.10), *p* = 0.365). In multivariate analysis, increasing age was associated with mortality (HR 1.01 (1.01–1.02), *p* < 0.001). Patients who underwent primary surgery had a lower risk of mortality relative to women who did not (HR = 0.50 (0.47–0.54, *p* < 0.001). Additionally, relative to patients with HR+/HER2− breast cancer, those with HER2+ disease had a lower risk of mortality (HR 0.86 (0.79–0.93), *p* < 0.001), and those with TN disease had a higher risk of mortality (HR 3.48 (3.22–3.76), *p* < 0.001).

### 3.2. Insurance Status

The demographic and clinical-pathological characteristics for the US cohort by insurance status in comparison to the AB cohort can be found in Table 1. There were significant differences in the distribution of the groups by age category (*p* = 0.006), year of diagnosis (*p* = 0.014), receipt of primary surgery (*p* < 0.001), and biomarker profile (*p* = 0.001). The US privately insured and US uninsured groups were slightly older relative to the US Medicaid and AB groups. Contribution to each group by year of diagnosis was more consistent in the US privately insured and US Medicaid groups but more variable in the US uninsured and AB groups. More patients in the US privately insured and AB groups underwent primary surgery compared with the US Medicaid and US uninsured groups. The distribution of the three US groups did not differ in terms of biomarker profile but all varied relative to the AB group as reported above. In the US groups, the proportion of patients with missing biomarker information was lowest for the US privately insured group and highest for the US uninsured group.

**Figure 1:**
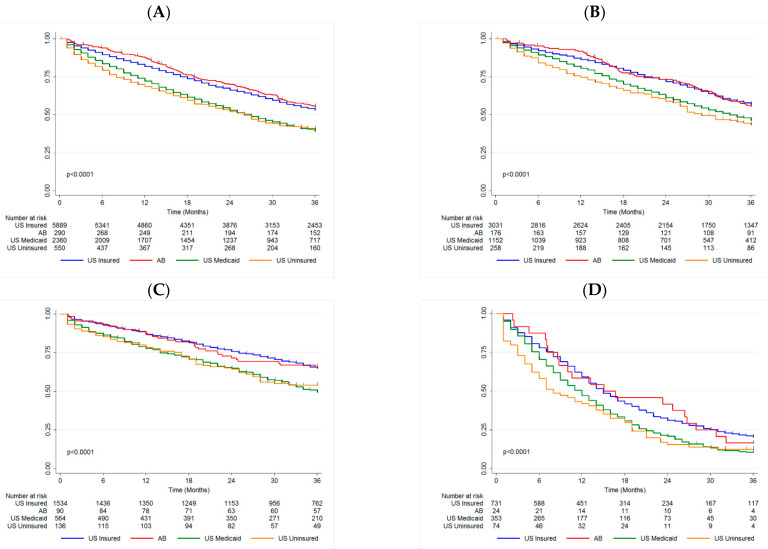
Kaplan-Meier with log-rank by Insurance Status in (**A**) Overall Cohort, (**B**) HR+/HER2− patients, (**C**) HER2+ patients, and (**D**) TN patients. AB Alberta, HR+ hormone receptor positive, HER2− human epidermal growth factor receptor-2 negative, HER2+ human epidermal growth factor receptor-2 positive, TN triple negative, US United States.

The median OS was not reached for the US privately insured and AB groups, and was 11 months and 8 months for the US Medicaid and US uninsured groups, respectively (*p* < 0.001, Figure 1A). The 3-year OS rates were comparable between US privately insured and AB groups (53.28% (51.95–54.59) and 55.54% (49.49–61.16), respectively). Both of these groups had improved survival relative to the US Medicaid and US uninsured groups (39.32% (37.25–41.37) and 40.53% (36.20–44.81)). In comparison to the US privately insured group, the AB cohort had similar survival (HR = 1.04 (0.87–1.25), *p* = 0.703), while both the US Medicaid (HR = 1.41 (1.32–1.52), *p* < 0.001) and US uninsured (HR = 1.44 (1.26–1.63), *p* < 0.001) groups had a higher likelihood of mortality (Table 2).

### 3.3. Insurance Status by Biomarker Profile

#### 3.3.1. HR+/HER2−

The median OS was not reached for the US privately insured and AB groups, and was 33 and 29 months for the US Medicaid and US uninsured groups respectively (*p* < 0.0001, Figure 1B). There was a significant difference in 3-year OS between both the US Medicaid and US uninsured groups (46.32% (43.27–49.31) and 43.42% (37.01–49.65)), relative to the US privately insured and AB groups (56.94% (55.08–58.75) and 55.68% (47.80–62.86)). This trend was maintained in the Cox model, adjusting for surgery and age (Table 2).

#### 3.3.2. HER2+

The median OS was not reached for the US privately insured, US uninsured or AB groups, and was 36 months for the US Medicaid group (*p* < 0.0001, Figure 1C). The US Medicaid and US uninsured groups had significantly worse 3-year OS (49.34% (44.91–53.61) and 53.97% (44.83–62.24)) compared to the US privately insured and AB groups (64.92% (62.37–67.34) and 65.83% (54.88–74.72)). This trend persisted in multivariate analysis (Table 2).

#### 3.3.3. TN

The median OS was 15 months for both the US privately insured and AB groups, while it was 12 months for the US Medicaid group and 8 months for the US uninsured group (*p* < 0.0001, Figure 1D). As for the other biomarker groups, the US Medicaid and US uninsured groups had significantly worse 3-year OS (10.74% (7.67–14.40) and 3.96% (6.05–21.37)) compared to the US privately insured and AB groups (20.56% (17.61–23.68) and 16.67% (5.22–33.70)). Again this trend persisted in multivariate analysis (Table 2). Unlike for the other biomarker groups, age was not associated with mortality for TN patients.

## 4. Discussion

In this retrospective, population-based cohort study, we found that overall survival for women with de novo MBC in the US and AB was similar. Considering health care coverage, the AB cohort had comparable survival to the US privately insured patients; however, US Medicaid and US uninsured patients had worse prognosis. In terms of biomarker profile, HER2+ patients fared best followed by HR+/HER2− and then TN. Increasing age was associated with worse prognosis only for those with HER2+ and HR+/HER2− disease. US privately insured and AB patients have a higher rate of surgery compared to US uninsured and US Medicaid patients. Furthermore, patients who underwent primary surgery fared better in comparison to those who did not. This improved outcome might be attributable to a lead time bias, related to the incidental detection of metastatic disease from use of routine staging investigations, rather than a beneficial effect of the surgery. In fact, a recent phase 3 randomized trial of the ECOG-ACRIN Research Group (E2108) demonstrated that early local therapies with surgery and radiotherapy do not improve overall survival in de novo metastatic breast cancer patients [14].

The identification of modifiable factors that influence the likelihood of survival among patients with metastatic BC is critical in improving patient care and population outcomes. Insurance status is one of the factors identified in many studies that could explain variances in the outcomes observed in patients with de novo metastatic breast cancer. Notably, Vaz-Luis et al. showed that the odds of early death for uninsured de novo metastatic breast cancer patients treated were more than 2.5 times higher than for insured patients [15]. Furthermore, Ellis et al. showed that the mortality rate of US patients covered by Medicaid is higher than for those covered by a private insurer [16]. This suggests that public insurance presents some risks of care inadequacies. Is this applicable when public insurance is provided in the context of a single-payer system of a universal health care coverage such as in Canada? To our knowledge, our current work is the first population-based study that directly compares OS between de novo metastatic breast cancer patients treated in Canada and the US based on insurance status.

We observed significant disparities in OS by insurance status among patients less than 65 years. The AB cohort and US privately insured group demonstrated similar 3-year OS. However, OS for the US Medicaid and US uninsured groups were significantly inferior. This result persisted after adjusting for age, biomarker profile, and receipt of primary surgery. These real-world findings go against the common thinking associated with universal health care policies, mainly that patients receive suboptimal care in comparison to their privately insured counterpart.

In our study, only patients less than 65 years old were compared according to health care insurance because of the lack of reliable insurance status in the SEER registry for older patients. Despite this limitation, we believe that the studied population is relevant and critical in the evaluation of key cancer care indicators. This group constitutes the workforce of a society. Generally with favourable performance status and co-morbidity profile, this younger population is more likely than an older population to be able to qualify, receive and tolerate the best standard of care. Therefore, using a younger population might compensate for one of our study’s deficiencies, that OS results are not adjusted for co-morbidities. We can easily conceive that women who are inadequately insured might also have numerous other serious or advanced co-morbid diseases due to a lack of health care access. This might affect the OS outcome defined as death from any cause. In fact, Fallahpour et al. demonstrated that moderate comorbidity defined by Charlson Comorbidity Index score 1 or 2, and severe comorbidity defined by Charlson Comorbidity Index score 3 or more, correlated with increased risk of breast-cancer related death [17]. Using breast cancer-specific survival instead of overall survival might overcome this limitation.

Other than co-morbid conditions, our study did not take into account socio-economic status. For the US cohort, an individual’s socio-economic status might be partly reflected by insurance status as US uninsured and US Medicaid patients are likely to have a lower income [2]. Many studies, performed on Canadian or US populations, demonstrated that lower socio-economic status correlates with worse breast cancer outcomes. In a study from Ontario, this factor appears to be associated to the OS outcomes independently of the treatment administered [18].

The racial disparity between the Canadian and US population is another factor that could confound this survival analysis. Ethnicity was not included as an independent variable in our study because this data is not recorded in the BDM repository. In the US, almost 13% of the population is African American as opposed to 3.5% in Canada [19,20]. Cumulative evidence suggests that the survival gap between African descendants and European descendants with cancer is, in part, due to biological difference. Many population-based retrospective cohort studies reported worse survival in people with an Afro-American ancestry despite adjusting for socio-economic factors, insurance status and treatments [21,22]. Furthermore, the prevalence of the triple negative breast cancer subtype is higher among African-American women [23]. The paradigm that triple negative breast cancer in African descendants has a more aggressive biology than in European descendants remains to be further elucidated [21]. Interestingly, within the triple negative subtype, it was shown by Vaz-Luis et al. that the correlation between insurance status and early death is even stronger: about 50% of uninsured patients will succumb to their cancer within 6 months as opposed to 35% of insured patients [15].

A nationwide cancer registry such as the SEER database is not available in Canada. In our study, a single provincial cohort was used. The selected cohort is not necessarily representative of the total Canadian population as provincial disparities with regards to accessibility to therapy must be considered [12]. For example, in Québec, some patients might have access to a drug through a program called Mesure du patient d’exception which allows physicians to request a special treatment for an exceptional case. The SEER database population also has some limitations in terms of representativeness of the US general population. This database has a slightly higher proportion of foreign-born people than the US general population. However, the education and poverty levels are comparable [24].

In the US, the ACA’s implementation led to Medicaid and private insurance expansion [25]. This study was performed within four years of the ACA’s implementation and coverage for the US population was still in a state of flux. This is reflected by the decrease in the number of US uninsured patients in 2014 [6]. A wide spectrum of private insurance plans is still available. High-deductible health plans that entail significant out-of-pocket costs have become the main private health policy type and are expected to gain in popularity with the ACA implementation. It has been shown that breast cancer patients covered with high-deductible health plans experienced delays in diagnosis and treatment [26]. In our study, we evaluated private insurance plans as a whole and we did not assess survival outcomes based on their individual coverage characteristics. Within the US privately insured group, significant differences might exist regarding access to certain drugs and delays in diagnosis and treatment initiation [7,25,26,27].

It remains unsure if the Medicaid insurance expansion as part of the ACA implementation will be able to rectify some of the disparities observed in cancer outcomes according to insurance status in US. In our study US privately insured and AB patients had similar OS, and US Medicaid and US uninsured patients had inferior OS. Lack of information on systemic therapy does not allow to draw a direct correlation between the inferior OS observed for US Medicaid and US uninsured patients and restricted access to different treatment options. Future studies that account for co-morbid health conditions, socio-economic status, ethnicity and scope of insurance plan are warranted to elucidate the trends observed.

## 5. Conclusions

This study provides evidence that a universal health care system based on a single public payer model is not inferior to a private insurance-based model for patients with de novo stage IV breast cancer. Our findings lend support to a universally accessible, single payer health care system.

## Figures and Tables

**Table 1:** Comparison of US Cohort by Insurance Status and AB Cohort.

	US Privately Insured		AB		US Medicaid		US Uninsured		
	***n***	**(%)**	***n***	**(%)**	***n***	**(%)**	***n***	**(%)**	***p*** **-Value**
	**6124**	**100.0%**	**291**	**100.0%**	**2493**	**100.0%**	**637**	**100.0%**	
**Age**									
Median	54		52		53		55		
Range	(19–64)		(22–64)		(20–64)		(21–64)		
**Age Group**									
18–49	1997	32.6%	105	36.1%	884	35.5%	186	29.2%	**0.006**
50–64	4127	67.4%	186	63.9%	1609	64.5%	451	70.8%	
**Year of Diagnosis**									
2010	1200	19.6%	50	17.2%	495	19.9%	114	17.9%	**0.014**
2011	1220	19.9%	65	22.3%	508	20.4%	136	21.4%	
2012	1205	19.7%	47	16.2%	481	19.3%	133	20.9%	
2013	1221	19.9%	64	22.0%	493	19.8%	160	25.1%	
2014	1278	20.9%	65	22.3%	516	20.7%	94	14.8%	
**Surgical Status**									
No Surgery	3678	60.1%	175	60.1%	1685	67.6%	487	76.5%	**<0.001**
Surgery	2312	37.8%	116	39.9%	750	30.1%	138	21.7%	
	134	2.2%	0	0.0%	58	2.3%	12	1.9%	
**Biomarker Profile**									
HR+/HER2−	3095	50.5%	176	60.5%	1183	47.5%	285	44.7%	**0.001**
HER2+	1589	25.9%	90	30.9%	590	23.7%	150	23.5%	
TN	760	12.4%	25	8.6%	369	14.8%	83	13.0%	
Missing	680	11.1%	0	0.0%	351	14.1%	119	18.7%	

AB Alberta, HR+ hormone receptor positive, HER2− human epidermal growth factor receptor-2 negative, HER2+ human epidermal growth factor receptor-2 positive, TN triple negative, US United States.

**Table 2:** Cox Multivariate Model by Insurance Status in Overall Cohort, HR+/HER2− Patients, HER2+ Patients, and TN Patients.

Overall Cohort	Multivariate		
	HR	95%CI	***p*** **-Value**
**US Privately Insured**	Reference		
AB	1.04	(0.87–1.25)	0.703
US Medicaid	1.41	(1.32–1.52)	<0.001
US Uninsured	1.44	(1.26–1.63)	<0.001
**No Surgery**	Reference		
Surgery	0.51	(0.47–0.55)	<0.001
**Age**			
(continuous)	1.01	(1.01–1.02)	<0.001
**HR+/HER2−**	Reference		
HER2+	0.87	(0.81–0.94)	0.001
TN	3.48	(3.21–3.76)	<0.001
**HR+/HER2−**		**Multivariate**	
	**HR**	**95%CI**	***p*** **-Value**
**US Privately Insured**	Reference		
AB	1.06	(0.84–1.35)	0.603
US Medicaid	1.36	(1.23–1.51)	<0.001
US Uninsured	1.42	(1.19–1.70)	<0.001
**No Surgery**	Reference		
Surgery	0.52	(0.47–0.57)	<0.001
**Age**			
(continuous)	1.01	(1.01–1.02)	<0.001
**HER2+**		**Multivariate**	
	**HR**	**95%CI**	***p*** **-Value**
**US Privately Insured**	Reference		
AB	0.93	(0.64–1.35)	0.701
US Medicaid	1.61	(1.39–1.87)	<0.001
US Uninsured	1.37	(1.04–1.81)	0.023
**No Surgery**	Reference		
Surgery	0.49	(0.42–0.56)	<0.001
**Age**			
(continuous)	1.03	(1.02–1.04)	<0.001
**TN**		**Multivariate**	
	**HR**	**95%CI**	***p*** **-Value**
**US Privately Insured**	Reference		
AB	1.06	(0.67–1.65)	0.814
US Medicaid	1.34	(1.16–1.54)	<0.001
US Uninsured	1.50	(1.15–1.95)	0.003
**No Surgery**	Reference		
Surgery	0.51	(0.45–0.58)	<0.001
**Age**			
(continuous)	1.00	(1.00–1.01)	0.220

AB Alberta, HR hazard ratio, CI confidence interval, HR+ hormone receptor positive, HER2− human epidermal growth factor receptor-2 negative, HER2+ human epidermal growth factor receptor-2 positive, TN triple negative, US United States.

## Data Availability

The data obtained from SEER and Alberta Health Services (AHS); Cancer Control Breast Data Mart (BDM) can be shared with the journal for review, if needed.

## Supplementary Materials

The following supporting information can be downloaded at: https://www.mdpi.com/article/10.3390/curroncol29010034/s1, Table S1: Comparison of US and AB Cohorts.
