# Supplementary material for: Survival in Women with De Novo Metastatic Breast Cancer: A Comparison of Real-World Evidence from a Publicly-Funded Canadian Province and the United States by Insurance Status
Source: Curr Oncol. 2022 Jan 16;29(1):383–91. doi: 10.3390/curroncol29010034 (PMC8774867; doi:10.3390/curroncol29010034)
Supplement: Supplementary file 1 [file curroncol-29-00034-s001.zip › curroncol-1538669-supplementary.pdf]

Table S1: Comparison of US and AB Cohorts

	US		AB		
	n	(%)	n	(%)	**p-value**
	**9,492**	**100.0%**	**291**	**100.0%**	
**Age**					
Median	54		52		**0.029**
Range	(19-64)		(22-64)		
**Age Group**					
18-49	3,132	33.0%	105	36.1%	0.270
50-65	6,360	67.0%	186	63.9%	
**Year of Diagnosis**					
2010	1,856	19.6%	50	17.2%	0.381
2011	1,904	20.1%	65	22.3%	
2012	1,864	19.6%	47	16.2%	
2013	1,930	20.3%	64	22.0%	
2014	1,938	20.4%	65	22.3%	
**Surgical Status**					
No Surgery	6,030	63.5%	175	60.1%	0.090
Surgery	3,254	34.3%	116	39.9%	
Missing	208	2.2%	0	0.0%	
**Biomarker Profile**					
HR+/HER2-	4,673	49.2%	176	60.5%	**0.012**
HER2+	2,378	25.1%	90	30.9%	
TN	1,228	12.9%	25	8.6%	
Missing	1,213	12.8%	0	0.0%	

AB Alberta, HR+ hormone receptor positive, HER2+ human epidermal growth factor receptor-2 positive, HER2- human epidermal growth factor receptor-2 negative, TN triple negative, US United States
